# The Role of Snake Venom Disintegrins in Angiogenesis

**DOI:** 10.3390/toxins16030127

**Published:** 2024-03-01

**Authors:** Patricia Bianca Clissa, Maisa Splendore Della-Casa, Bianca Cestari Zychar, Sabri Saeed Sanabani

**Affiliations:** 1 Immunopathology Laboratory, Butantan Institute, São Paulo 05585-090, Brazil; maisa.casa@butantan.gov.br; 2 Physiopathology Laboratory, Butantan Institute, São Paulo 05585-090, Brazil; bianca.zychar@butantan.gov.br; 3 Laboratory of Medical Investigation LIM-56, Division of Dermatology, Medical School, University of São Paulo, Sao Paulo 05508-220, Brazil

**Keywords:** snake venom, angiogenesis, integrins, metalloproteinases, disintegrins

## Abstract

Angiogenesis, the formation of new blood vessels, plays a critical role in various physiological and pathological conditions. Snake venom disintegrins (SVDs) have been identified as significant regulators of this process. In this review, we explore the dual roles of SVD in angiogenesis, both as antiangiogenic agents by inhibiting integrin binding and interfering with vascular endothelial growth factors and as proangiogenic agents by enhancing integrin binding, stimulating cell migration and proliferation, and inducing neoangiogenesis. Studies in vitro and in animal models have demonstrated these effects and offer significant therapeutic opportunities. The potential applications of SVD in diseases related to angiogenesis, such as cancer, ocular diseases, tissue regeneration, wound healing, and cardiovascular diseases, are also discussed. Overall, SVDs are promising potential therapeutics, and further advances in this field could lead to innovative treatments for diseases related to angiogenesis.

## 1. Introduction

### 1.1. Angiogenesis

Angiogenesis is a complex biological process involving the formation of new blood vessels from preexisting vessels [1]. It plays a crucial role in various physiological and pathological conditions, including embryogenesis, wound healing, and tumor growth [2]. The process of angiogenesis is tightly regulated by multiple signaling pathways and factors. Matrix metalloproteinases are primarily responsible for degrading the basement membrane surrounding existing blood vessels, allowing endothelial cells to migrate and proliferate toward the angiogenic stimulus [3]. One of the key proangiogenic factors is vascular endothelial growth factor (VEGF), which is essential for the formation of new blood vessels during embryonic development and is produced by various cell types, including tumors [4]. VEGF binds to specific receptors on endothelial cells, promoting their survival, migration, and differentiation [5,6]. Other factors involved in angiogenesis regulation include fibroblast growth factors (FGFs), platelet-derived growth factor (PDGF), and angiopoietins [3]. These factors act synergistically to ensure the proper formation and remodeling of blood vessels. The regulation of angiogenesis is maintained through a dynamic balance between proangiogenic and antiangiogenic factors. This balance can be disrupted in various diseases, leading to either excessive or insufficient blood vessel formation [1]. Insufficient angiogenesis, on the other hand, can lead to tissue ischemia, impaired wound healing, and various cardiovascular diseases [2]. Peripheral artery disease (PAD) is an example where the narrowing or blockage of blood vessels reduces blood flow to the legs or arms, resulting in pain, skin ulcers, and an increased risk of amputation [2]. Therapeutic angiogenesis, which aims to promote blood vessel formation, has been investigated as a potential treatment approach for PAD, with the administration of VEGF or the use of gene therapy [1].

It is important to emphasize that integrins, a group of transmembrane proteins that play a crucial role in cell adhesion and communication between cells and the extracellular environment, are the focus of interest, as they play an important role in angiogenesis [7].

### 1.2. Integrins

Integrins play an essential role in the regulation of biological processes such as cell migration, adhesion, proliferation, differentiation and signaling [8]. Integrins are heterodimeric glycoproteins consisting of alpha and beta subunits, which together form a complex transmembrane receptor [9]. A total of 18 alpha subunits and eight beta subunits have been identified in mammalian cells, allowing the formation of 24 different heterodimers. The α subunit determines affinity to the extracellular matrix component (ECM), while the β subunit associates with cytoplasmic structural and regulatory proteins. These subunits have a long transmembrane domain and a short cytoplasmic domain associated with cytoskeletal proteins [10]. Although integrins are constitutively expressed on the cell surface, they need to be activated to interact with their ligands [11]. Such activation can occur in the presence of chemokines and cytokines and is characterized by a conformational change at the extracellular integrin domain that exposes their binding sites on the α and β subunits, allowing them to interact with their ligands on the ECM or with proteins on the membranes of neighboring cells. This interaction is primarily controlled by a conserved tripeptide pattern of arginine, glycine, and aspartate, commonly known as the Arg-Gly-Asp motif (RGD) [12,13,14]. They are crucial for cell adhesion, migration, and signal transduction by mediating interactions between cells and ECM proteins. The main role of these molecules is to provide a link between the cytoskeleton of the cell and certain ECM components, such as fibronectin, vitronectin, laminin, and collagen. In addition, they are responsible for triggering intracellular signal transduction pathways upon interaction with the ECM. Integrins can undergo conformational changes that influence their ligand binding properties and downstream signaling events [15]. They possess a unique arrangement of cysteine residues that enables them to adopt a compact and stable structure consisting of a well-defined loop disulfide array [16]. This structural motif is critical for their high-affinity binding to integrins as it allows them to bind to specific integrin subunits and block their ligand binding sites. In addition, integrins also interact with growth factor receptors to regulate cell migration, blood vessel development, and angiogenesis. Importantly, the understanding of the mechanism of action of integrins paralleled the discovery of proteins from snake venoms, called disintegrins, which act as potent inhibitors of platelet aggregation and integrin receptor-dependent cell adhesion [17,18].

### 1.3. Snake Venom Metalloproteinases and Snake Venom Disintegrins

**Figure 1:**
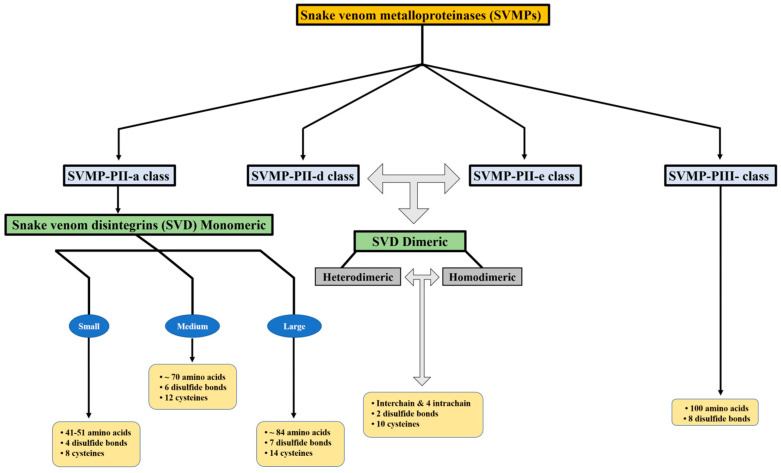
Disintegrins are categorized according to their structural composition. The number of disulfide bonds and length of the polypeptide chain determine this categorization.

Snake venom metalloproteinases (SVMPs) are a major component of most crotalid and viperid venoms [19]. SVMPs are known to be a class of key toxins involved in the pathophysiology associated with viperid venoms and are classified into classes and subclasses from P-I to P-III according to the organization of their domains, as shown in Figure 1 [20]. In general, class PI includes metalloproteinases that have only a catalytic domain containing zinc; class PII, metalloproteinases that have a catalytic domain followed by a disintegrin domain containing the tripeptide RGD (arginine-glycine-aspartic acid); and class PIII, metalloproteinases that have a catalytic domain, a disintegrin-like domain, and a cysteine-rich domain [21].

**Figure 2:**
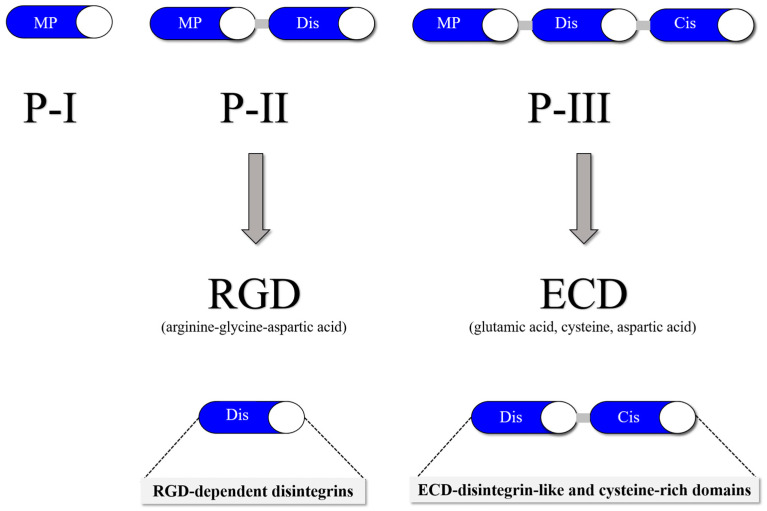
SVMP classification is divided into three classes: P-I (20–30 kDa), which in its mature form contains a metalloproteinase domain; P-II (30–60 kDa), which contains a disintegrin domain linked to the C-terminus of the metalloproteinase domain; and P-III (60–100 kDa), which consists of a metalloproteinase domain, a disintegrin-like domain and a cysteine-rich domain.

Snake venom disintegrins (SVDs) are a class of proteins derived from SVMPs. The disintegrins present in snake venoms can be formed in the venom gland in two distinct ways: (1) By proteolysis of SVMPs of class P-II, where cleavage occurs between the catalytic domain and the disintegrin domain, leaving only the disintegrin domain. These disintegrins are known as RGD-dependent disintegrins, which are abundant in viperid venoms and contain the sequence XGD (X-Gly-Asp), MLD (Met-Leu-Asp), or K/RTS (Lys/Arg-Thr-Ser) on the exposed surface of the loop that specifically binds to integrins on the surface of different cell types [22,23]. The amino acid sequences immediately adjacent to the RGD site of disintegrins could form an extended RGD locus that, in conjunction with the conformational representation of the RGD sequence, could be involved in determining integrin selectivity and affinity [13]. The family of disintegrins containing the RGD motif is widely recognized as the most extensive and well-studied group. Most of these units function as monomers (small, medium, or large), but a subgroup has the ability to combine into dimers and form homo- or heterodimers [24]. (2) Proteolysis of class P-III SVMPs results in fragments that covalently link the disintegrin-like and cysteine-rich domains and are referred to as ECD-disintegrin-like/cysteine-rich domains (Figure 2). This disintegrin-like domain has a sequence of non-RGD tripeptides in its binding site [21,25].

SVDs are known for their ability to bind to integrin receptors and modulate various cellular functions, such as inflammation, apoptosis in endothelial cells and inhibition of platelet aggregation [24]. Jararhagin-C, an SVD containing the ECD-disintegrin-like/cysteine-rich domains produced by the proteolytic cleavage of Jararhagin (SVMP P-III), is present in *Bothrops jararaca* venom and specifically interacts with α2β1 integrin, inhibiting collagen and ADP-induced platelet aggregation [8]. Jararhagin-C is also capable of triggering the local release of cytokines [26] and induces changes in leukocyte–endothelium interactions through the expression of the adhesion molecules ICAM-1, CD11a and CD11b [27,28]. Alternagin-C, a toxin isolated from *B. alternatus* venom, is a protein composed of the ECD-disintegrin-like/cysteine-rich domain, with 92% homology to Jar-C. It is able to interfere with α2β1 integrin functions and may contribute to apoptosis by interfering with cell adhesion [29].

One of the notable characteristics of SVDs is their ability to inhibit platelet aggregation, an important step in blood clot formation [30]. This property has led to the exploration of disintegrins as potential antithrombotic drugs for the prevention and treatment of conditions such as deep vein thrombosis and stroke. For example, Insularin, a monomeric RGD-disintegrin isolated from the venom of *Bothrops insularis*, strongly inhibits human platelet aggregation and fibrinogen adhesion of endothelial cells [31]. Echistatin, an RGD disintegrin from the venom of the saw-scaled viper (*Echis carinatus*), effectively inhibits platelet aggregation by binding to integrin αIIbβ3 [32]. Upon binding to integrins, SVDs interfere with various cellular processes. Disintegrins have gained attention in the field of medicine due to their unique properties and potential therapeutic applications.

SVDs have also shown promise in cancer research [33,34,35]. Integrins play a crucial role in tumor growth, invasion, and metastasis [36]. By targeting specific integrins expressed on cancer cells, SVD can potentially inhibit the proliferation and migration of these cells. Contortrostatin, a disintegrin from the venom of the *Agkistrodon contortrix contortrix*, has been found to have anticancer properties by interacting with integrins αvβ3 and α5β1 [37]. Vicrostatin, a recombinant disintegrin developed by fusing 62 N-terminal amino acids of the disintegrin Contortrostatin with 6 C-terminal amino acids of Echistatin, is the best-characterized and most preclinically advanced disintegrin shown to target multiple tumor-associated integrins and to exhibit potent antitumor and antiangiogenic activity in in vitro and in vivo models without appreciable toxicity [23].

Since the 1987 report on the isolation of trigramin, the first disintegrin isolated from the venom of *Trimeresurus gramineus*, approximately 100 other disintegrins have become known from snake venoms with potential applications in cancer research and therapy (see the list in [17]).

### 1.4. The Role and Properties of SVDs in Angiogenesis

One of the most well-known effects of SVD is its ability to prevent cell adhesion to extracellular matrix proteins [16]. By occupying the ligand binding sites on integrins, disintegrins disrupt the attachment of cells to their surrounding microenvironment. This disruption can have profound effects on cell migration, invasion and angiogenesis, processes that are essential for tumor progression and metastasis [18]. Dysregulation of integrin activity has been implicated in numerous pathological conditions, such as cancer, inflammation, and thrombosis [38,39]. Consequently, inhibiting integrin binding has emerged as a promising therapeutic strategy [40,41].

Several studies highlight the dual role of SVD in angiogenesis, with some disintegrins acting as inhibitors and others acting as enhancers of the process [42,43,44,45]. The interaction between disintegrins and integrin receptors on endothelial cells plays a critical role in modulating angiogenesis. Therefore, inhibitors of α1β1 and α2β1 integrins alone or in combination with antagonists of other integrins involved in angiogenesis (eg. αvβ3, αvβ5, αIIbβ3 and α6β4) may prove beneficial in controlling neovascularization, making SVDs valuable tools for the study and potential manipulation of this complex biological process.

Some SVDs have been identified as potent inhibitors of angiogenesis (Table 1). For instance, obtustatin, a disintegrin isolated from the venom of *Vipera lebetina obtusa*, has been shown to selectively inhibit α1β1 integrin, leading to the suppression of angiogenesis in vitro and in vivo [46]. A similar effect was also observed with viperistatin isolated from the venom of *Vipera paleastinae* [47]. Furthermore, jerdostatin, isolated from the venom of *Trimeresurus jerdonii* [48], and lebestatin, isolated from the venom of *Macrovipera lebetina* [42], two other small monomeric disintegrins that antagonize the function of the α1β1 integrin, have also been described as inhibitors of angiogenesis. Rhodostomin, a medium disintegrin from the venom of *Calloselasma rhodostoma*, has been reported to inhibit angiogenesis by binding to integrins and inhibiting bFGF-induced proliferation of endothelial cells [49].

On the other hand, certain SVDs have been found to promote angiogenesis. For instance, Jararhagin-C has been shown to promote angiogenesis by activating integrin receptors and stimulating endothelial cell migration, increasing the density of blood vessels and the synthesis of proangiogenic cytokines (VEGF and FGF) [44].

Alternagin-C exhibits both pro- and antiangiogenic effects depending on the concentration. Concentrations less than 50 nM were found to be proangiogenic, whereas concentrations greater than 100 nM were found to be antiangiogenic both in vitro and in vivo [50,51]. Alternagin-C inhibits VEGF/VEGFR2 signaling after binding to α2β1 integrin, resulting in impaired angiogenesis [50].

#### Interference of SVD with VEGF

VGEF is a key molecule for regulating and promoting the angiogenesis process through the stimulation of proliferation, migration and organization of endothelial cells, triggering signaling cascades such as Notch, angiopoietin/Tie, MAPK, FAK, PI3K/AKT, ERK1/2, Src and PLCγ. While VEGFs stimulate the proliferation of endothelial cells, integrins help anchor these cells to the extracellular matrix to ensure that vessel formation occurs in an organized manner. SVDs can affect vascular VEGF, which plays a crucial role in regulating blood vessel formation and vascular permeability [58,59,60].

There are several ways in which SVDs can interfere with VEGF function. Disintegrins can inhibit angiogenesis, the process of forming new blood vessels, which is essential for tumor growth and metastasis [7,61]. By interfering with VEGF-induced signaling, disintegrins hinder the formation of new blood vessels and thus impair tumor progression [62]. Moreover, disintegrins can interfere with the signal transduction pathways initiated by VEGF [63,64]. This interference can affect various cellular processes, including gene expression and protein synthesis, which are crucial for the regulation of blood vessel formation and permeability [58]. Overall, SVD exerts its effects on VEGFs through the inhibition of angiogenesis and interference with signal transduction pathways, ultimately influencing the regulation of blood vessel formation and vascular permeability.

### 1.5. Anti-Angiogenic Effects of SVDs

Recent studies have demonstrated the antiangiogenic effects of SVD, suggesting their potential as therapeutic agents for various human diseases, including cancer. In vitro experiments have shown that contortrostatin exhibits antiangiogenic activity [37]. Similarly, DisBa-01, a disintegrin from *Bothrops alternatus* snake venom, has been found to inhibit the proliferation, migration, and tube formation of human umbilical vein endothelial cells, which play crucial roles in angiogenesis [54]. Echistatin has also been shown to inhibit the proliferation and migration of human microvascular endothelial cells [63]. Animal model studies have further supported the antiangiogenic effects of SVD. For instance, contortrostatin has been found to inhibit tumor growth and angiogenesis in a mouse model of melanoma [37,62]. Similarly, Echistatin has demonstrated the ability to inhibit tumor growth and angiogenesis in a mouse model of glioma [65]. Agkistin is a P-II class SVMP containing a metalloproteinase and a disintegrin domain purified from crude venom of Formosan Agkistrodon acutus. Agkistin-s is the disintegrin domain of Agkistin and induces endothelial cell apoptosis, exhibiting profound antiangiogenic activity [57].

Additionally, Leberagin-C (Leb-C), a disintegrin from *Macrovipera lebetina* transmediterrannea snakes, has been shown to disrupt the adhesion, migration, and invasion capabilities of MDA-MB-231 breast cancer cells and its highly metastatic D3H2LN subpopulation [52].

## 2. Potential Applications of SVD in Angiogenesis-Related Diseases

Diseases characterized by abnormal angiogenesis, such as cancer and ocular diseases, represent a major public health challenge worldwide [66]. The search for effective therapeutic agents to modulate angiogenesis is of great interest to medical researchers [67]. SVDs have emerged as potential candidates for antiangiogenic and proangiogenic therapies [17,23,68]. Some of the diseases are characterized by abnormal angiogenesis.

### 2.1. Cancer

Cancer is a multifaceted group of diseases that poses a major health challenge worldwide and results in a significant burden of morbidity and mortality. The etiology of cancer involves a multifaceted interplay between genetic and epigenetic elements that lead to alterations in the genome and subsequently trigger uncontrolled cell proliferation in the tissues and organs of the body [69]. Cancer can be classified into different types based on the specific cell type from which the tumor originates. These include carcinoma, sarcoma, lymphoma and leukemia, germ cell tumor and blastoma [70]. Worldwide cancer statistics show that in 91 of 172 countries, cancer is the primary or secondary leading cause of death before the age of 70. In 22 other countries, cancer is the third or fourth leading cause of death [71]. Chemotherapy, radiotherapy, immunotherapy and surgery together form an integral part of modern cancer treatment. The pharmaceutical industry is currently conducting extensive research to develop innovative drugs of natural origin to mitigate the adverse effects associated with cancer treatments [72,73,74]. Tumor angiogenesis, the sprouting of new blood vessels into tumors, is vital for sustained tumor growth, progression, and metastasis [75,76]. Various growth factors and cytokines orchestrate the angiogenic process, including VEGF and FGF [77,78]. The abnormal angiogenesis associated with cancer provides an opportunity for therapeutic intervention by targeting angiogenic factors or inhibiting the endothelial cell response [79,80]. Changes in the structure of tumor vessels toward a more mature phenotype could also promote resistance. Therefore, the development of drug resistance is important in the development of new antiangiogenic therapies [7].

**Figure 3:**
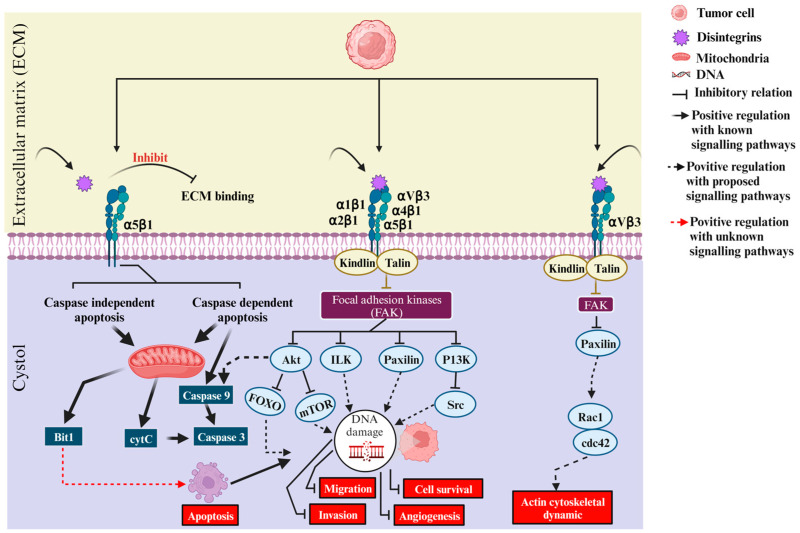
This figure shows how disintegrins act on different integrins and initiate intracellular signaling in cancer cells.

A chimeric recombinant disintegrin called Vicrostatin (VCN) has been used in a number of preclinical studies in various tumor models. VCN is a monomer prepared by recombinant modification of the primary Contortrostatin sequence by replacing the C-terminal tail with 6 aa derived from the C-terminus of Echistatin. It retained the targeting of Contortrostatin to αIIbβ3-, α5β1, αvβ3, and αvβ5 integrins but additionally showed more than 10-fold higher affinity for α5β1-integrin. Compared with the homodimeric structure of contortrostatin, the novel chimeric disintegrin VCN was active as a monomer, which allowed its production in larger amounts than several other cloned and expressed disintegrins. Like Contortrostatin, VCN has been shown to inhibit tumor cell adhesion, endothelial and tumor cell invasion, and angiogenesis. This chimeric recombinant toxin has shown promising results in preclinical experimental models of breast cancer, prostate cancer, ovarian cancer and glioblastoma [23]. Figure 3 shows the intracellular reactions that take place when disintegrins bind to integrin targets, as illustrated in the current literature. The involvement of α5β1-, αVβ3-, α1β1-, α2β1-, α4β1-, and α5β1-integrins in survival, proliferation, infiltration, motility, cytoskeletal reorganization, angiogenesis, apoptosis, and interaction with snake venom disintegrins triggers the onset of these mechanisms in cancer cells [81].

### 2.2. Ocular Diseases, e.g., Diabetic Retinopathy

Diabetic retinopathy (DR), a common microangiopathic sequela of diabetes mellitus [82], has a significant impact worldwide. It affects more than 100 million people and is a major contributor to visual impairment and blindness in industrialized countries [83]. DR is characterized by abnormal neovascularization in the retinal tissue of diabetic patients, leading to impaired visual function and eventual loss of vision [84]. The etiology of DR is primarily defined by the simultaneous occurrence of neurovascular unit dysfunction, blood-retinal barrier disruption, inflammatory processes, capillary nonperfusion or ischemia, and neoangiogenesis [85]. The disease is primarily driven by increased VEGF levels in response to ischemic or hypoxic stimuli, causing several alterations at different levels and subsequent neovascularization [86,87]. Pathological angiogenesis in DR disrupts retinal function and causes visual complications. It has been reported that various fractions in snake venom markedly increase insulin secretion without causing harmful effects. For example, a number of disintegrin isoforms were identified in *Crotalus vegrandis* venom and confirmed through partial sequencing as being homologous to various other snake disintegrins that share the active RGD motif near the C-terminus [88]. These snake venom disintegrins were shown to significantly enhance insulin secretion from BRIN-BD11 cells, suggesting disruption of the cellular signaling pathways activated by integrins, including receptor tyrosine kinases. These fractions have the potential to positively influence insulin secretion [88].

## 3. Utilization of SVD as Pro-Angiogenic Agents

While disintegrins are usually known for their antiangiogenic properties, recent research has also revealed their potential as proangiogenic agents. Alternagin-C (ALT-C) is an ECD-containing disintegrin-like/cysteine-rich disintegrin isolated from the venom of the snake *Rhinocerophis alternatus* that induces endothelial cell proliferation and angiogenesis both in vitro and in vivo by upregulating the expression of VEGF and its receptors [89]. ALT-C binds to the major collagen receptor α2β1 integrin, inhibiting cell adhesion to collagen, triggering downstream signaling molecules, and inducing a significant increase in several genes related to cell cycle control (VEGF, inducible early growth response, interleukin 11, early growth response 2 and 3, and the insulin-induced gene) [51]. ALT-C also induced significant cytoskeleton dynamic changes with the polymerization of F-actin, focal adhesion kinase (FAK), and phosphoinositol 3-kinase (PI3K) activation, as well as erk-2 translocation [90]. ALT-C induced the formation of new vessels, and the expression of VEGF in the injured tissue indicated the usefulness and effectiveness of ALT-C as a proangiogenic disintegrin-like protein [45].

### 3.1. Tissue Regeneration and Wound Healing

Wound healing and regeneration are multifaceted biological phenomena that occur throughout the human lifespan. After an injury, various cellular processes are immediately initiated and coordinated to initiate a response [91]. Improper repair procedures can cause this process to be delayed, with immediate consequences for the individual, including physical discomfort, impaired rehabilitation progress, limb amputation, and in the most severe cases, death from septicemia [92]. Like other natural healing processes, the repair mechanism also relies on the coordination of different cell activities vital for recovery. These activities encompass cell survival, growth, movement, and the creation of new cells. They are orchestrated through cellular interactions with both the extracellular matrix (ECM) that surrounds them and with other neighboring cells. These interactions are made possible by specialized receptors found on cell membranes, which belong to the integrin family [93]. A recent study led by Ferreira and colleagues [94] used an in vivo model with subcutaneous sponge implants to investigate the potential of jararhagin-C (Jar-C). Their goal was to understand how Jar-C might stimulate collagen deposition and the production of important soluble substances, including VEGF and transforming growth factor beta-1 (TGFβ-1). These substances play important roles in processes such as angiogenesis and fibrogenesis, which are closely associated with tissue repair. The results of the study suggest that Jar-C may have positive effects on tissue repair by promoting these natural responses in the body. Moreover, Alternagin-C is also the subject of research. Within 7 days, this cysteine-rich, disintegrin-like protein accelerates wound healing in rats by increasing type I collagen deposition and fibroblast density and reducing inflammation [95]. In another study, Rabelo et al. [96] revealed that ALT-C increased collagen synthesis in mouse fibrovascular tissue. From the above results, it can be concluded that SVD has the ability to promote angiogenesis by modulating endothelial cell behavior, facilitating cell migration, and upregulating proangiogenic factors. However, further research is needed to gain a comprehensive understanding of the underlying mechanisms and to improve the therapeutic application of disintegrins in the context of tissue regeneration and wound healing.

### 3.2. Cardiovascular Diseases

Integrins have a considerable influence on the development of cardiac fibrosis. The diseased heart exhibits altered expression and integrin functions [97]. Targeting integrins and their associated proteins can be a potential therapeutic target for myocardial fibrosis. Certain disintegrins have been extensively researched and subsequently approved by the Food and Drug Administration (FDA), making them viable pharmaceutical agents in modern medicine. Tirofiban, marketed as Aggrastat^®^, is a synthetic pharmaceutical agent developed by Medicure International, Inc. of Winnipeg, MB, Canada. This drug is derived from the RGD domain found in Echistatin [98]. In addition, this compound undergoes a chemical change that increases its affinity for platelet glycoproteins, especially GPIIb/IIIa receptors [32]. Therefore, this drug is able to prevent platelet aggregation and other thrombotic activities by competing with fibrinogen for the RGD domain recognition site in the GPIIb/IIIa receptor [98,99,100]. In 1998, the Food and Drug Administration (FDA) approved tirofiban as a therapeutic intervention for acute coronary syndrome [101]. Additionally, in 1998, the FDA approved eptifibatide (Integrilin^®^, Millennium Pharmaceuticals, Inc., Cambridge, MA, USA), an alternative molecule to inhibit platelet aggregation. Schering-Plough subsequently acquired license rights to this drug in 2005 [102]. The development of this active substance took place in parallel with research into synthetic peptide analogs of barbourin, a disintegrin from *Sistrurus miliarius barbouri* [103].

Cardiovascular diseases, including acute myocardial infarction, coronary artery disease, endothelial dysfunction, and chronic ischemia, are considered one of the leading causes of death worldwide [104]. Therefore, there is great interest in pharmaceutical agents that offer a new and effective therapeutic approach to improve the functionality of the myocardium and/or facilitate its regeneration after injury. Platelets have been studied extensively because of their crucial role in primary hemostasis, the body’s initial response to arterial injury. Only recently, however, have we begun to study their contribution to immunological processes, cardiovascular disease, cancer, and various other pathological conditions [105,106,107]. The glycoprotein receptor GPIIbIIIa has been extensively studied as a primary receptor on platelets for the functional effects of snake venom-derived disintegrins. Each individual platelet is equipped with approximately 80,000 GPIIbIIIa receptors located both on the plasma membrane and in the α-granules [108]. After activation, the number of these receptors increases significantly, facilitating the formation of a permanent hemostatic plug [81]. Certain SVDs, including those of *Agkistrodon piscivorus piscivorus* and *Echis carinatus sochureki*, namely, Applagin and Echistatin, show remarkable affinity for the RGD motif on resting platelets. This specific binding results in the potent inhibition of platelet aggregation. Consequently, SVDs have emerged as promising candidates for drug development to antagonize platelet integrins, demonstrating their pharmacological potential as both platelet aggregation inhibitors and antithrombotic agents. From a clinical perspective, these drugs can effectively reduce the likelihood of acute ischemic episodes and serve as preventive measures against thrombotic sequelae. RGD disintegrins have been extensively studied and are considered the largest family within this category [17,109]. A number of disintegrins have been extracted from snake venoms, particularly viper venom, and characterized as agents with antithrombotic properties [110]. For example, trigramin inhibits platelet aggregation in platelet-rich plasma triggered by adenosine diphosphate (ADP), collagen, or epinephrine [56]. The same disintegrin has been shown to inhibit platelet aggregation both in vitro and in vivo by preventing the binding of fibrinogen to platelets induced by agonists of aggregation, such as ADP-activated platelets [111].

By promoting angiogenesis, SVDs have shown promise in preclinical models of cardiac ischemia. Previous studies have shown that administration of ALT-C in a single dose after a period of 7–9 days resulted in an increase in cardiac muscle contractile force in fish [45,112]. This intervention also led to an upregulation of the expression of important proteins involved in calcium processing and an increase in the level of VEGF in the myocardium. In addition, administration of ALT-C stimulated angiogenesis and thus protected cardiomyocytes from the deleterious effects of negative tropism caused by hypoxia/reoxygenation. Evaluation of the safety and efficacy of disintegrins in promoting angiogenesis and improving cardiac function in patients with cardiovascular disease requires the conduct of clinical trials.

## 4. Conclusions

SVDs play a critical role in modulating angiogenesis by either inhibiting or promoting this process. Their mechanisms of action offer valuable insights into potential therapeutic applications for angiogenesis-related diseases. These SVDs show promise as potent antiangiogenic agents for the treatment of cancer and ocular diseases and as proangiogenic agents for tissue regeneration and wound healing. However, further research is needed to fully understand their molecular mechanisms and optimize their therapeutic potential. Further exploration of SVDs could lead to the discovery of new molecules or the development of synthetic analogs with improved stability and specificity. These advances have the potential to revolutionize the treatment of diseases related to angiogenesis and open new avenues for personalized medicine approaches targeting angiogenesis.

## Figures and Tables

**Table 1:** List of disintegrins found in snake venom that have pro- and/or anti-angiogenic properties.

Name (Source)	Recognizing Motif	Physiological Target	Angiogenic Factors	References
Jararhagin-C	ECD-disintegrin-like/cysteine-rich domains	Interferes with α2β1 integrin functions	Pro-angiogenic	[8]
Alternagina-C			Exhibits both pro- and anti-angiogenic effects	[29,50,51]
Leberagin-C	Disintegrin-like	Interferes with αvβ3, αvβ6, and α5β1 integrins	Anti-angiogenesis	[52]
Echistatin	RGD-dependent disintegrins	Binds to integrin αIIbβ3, GPIIb/IIIa and interacts with αvβ3 integrin	Anti-angiogenesis	[14,32,53]
Rhodostomin				[49]
Contortrostatin		Interacts with the integrins αvβ3 and α5β1		[37]
Vicrostatin		antagonize the function of the αIIbβ3, αvβ3, αvβ5 and α5β1 integrins		[23]
DisBa-01		Binds to integrin αvβ3		[54]
Aggretin		Binds to integrin α2β1	Pro-angiogenic	[55]
Trigramin		Binds to αIIbβ3, α8β1, αvβ3, αvβ5 and/or α5β1 integrins		[56]
Obtustatin	KTS-disintegrin	Selectively inhibit α1β1-integrin	Anti-angiogenesis	[46]
Viperistatin		Inhibitory activity against collagen receptors, α1β1 and α2β1-integrins		[47]
Lebestatin		Inhibis binding of α1β1 integrin to type IV and type I collagen		[42]
Jerdostatin	RTS-disintegrin	Antagonizes the function of the α1β1 integrin	Anti-angiogenesis	[48]
Agkistin-s		InteractS with GPIB	Anti-angiogenesis	[57]

## Data Availability

Not applicable.
